# Deep learning-based automatic delineation of the hippocampus by MRI: geometric and dosimetric evaluation

**DOI:** 10.1186/s13014-020-01724-y

**Published:** 2021-01-14

**Authors:** Kaicheng Pan, Lei Zhao, Song Gu, Yi Tang, Jiahao Wang, Wen Yu, Lucheng Zhu, Qi Feng, Ruipeng Su, Zhiyong Xu, Xiadong Li, Zhongxiang Ding, Xiaolong Fu, Shenglin Ma, Jun Yan, Shigong Kang, Tao Zhou, Bing Xia

**Affiliations:** 1 Department of Radiation Oncology, The Affiliated Hangzhou Hospital of Nanjing Medical University, Hangzhou, China; 2 Department of Radiation Oncology, Shanghai Chest Hospital, Shanghai Jiao Tong University, Shanghai, China; 3 Department of Radiation Oncology, Hangzhou Yikang Chinese Medicine Oncology Hospital, Hangzhou, China; 4 Department of Radiation Oncology, Key Laboratory of Clinical Cancer Pharmacology and Toxicology Research of Zhejiang Province, Affiliated Hangzhou Cancer Hospital, Hangzhou, China; 5 Department of Radiology, Key Laboratory of Clinical Cancer Pharmacology and Toxicology Research of Zhejiang Province, Affiliated Hangzhou First People’s Hospital, Hangzhou, China; 6 Beijing Allcure Medical Technology Group Co., Ltd., Beijing, China; 7 Shandong Cancer Hospital and Institute, Shandong First Medical University and Shandong Academy of Medical Sciences, Jinan, China

**Keywords:** Hippocampus, MRI, Artificial intelligence

## Abstract

**Background:**

Whole brain radiotherapy (WBRT) can impair patients’ cognitive function. Hippocampal avoidance during WBRT can potentially prevent this side effect. However, manually delineating the target area is time-consuming and difficult. Here, we proposed a credible approach of automatic hippocampal delineation based on convolutional neural networks.

**Methods:**

Referring to the hippocampus contouring atlas proposed by RTOG 0933, we manually delineated (MD) the hippocampus on the MRI data sets (3-dimensional T1-weighted with slice thickness of 1 mm, n = 175), which were used to construct a three-dimensional convolutional neural network aiming for the hippocampus automatic delineation (AD). The performance of this AD tool was tested on three cohorts: (a) 3D T1 MRI with 1-mm slice thickness (n = 30); (b) non-3D T1-weighted MRI with 3-mm slice thickness (n = 19); (c) non-3D T1-weighted MRI with 1-mm slice thickness (n = 11). All MRIs confirmed with normal hippocampus has not been violated by any disease. Virtual radiation plans were created for AD and MD hippocampi in cohort c to evaluate the clinical feasibility of the artificial intelligence approach. Statistical analyses were performed using SPSS version 23. *P* < 0.05 was considered significant.

**Results:**

The Dice similarity coefficient (DSC) and Average Hausdorff Distance (AVD) between the AD and MD hippocampi are 0.86 ± 0.028 and 0.18 ± 0.050 cm in cohort a, 0.76 ± 0.035 and 0.31 ± 0.064 cm in cohort b, 0.80 ± 0.015 and 0.24 ± 0.021 cm in cohort c, respectively. The DSC and AVD in cohort a were better than those in cohorts b and c (*P* < 0.01). There is no significant difference between the radiotherapy plans generated using the AD and MD hippocampi.

**Conclusion:**

The AD of the hippocampus based on a deep learning algorithm showed satisfying results, which could have a positive impact on improving delineation accuracy and reducing work load.

## Introduction

Brain metastases are an increasingly common complication of systemic cancers [1, 2]. Approximately 20–40% of the patients with primary extra-cranial cancer develop brain metastases during the course of their disease [3], which is usually associated with poor prognosis requiring urgent treatment [4]. Currently, brain radiotherapy (RT), including whole brain radiotherapy (WBRT) and stereotactic radiosurgery (SRS), is still one of the most important treatments [5].

However, patients receiving brain RT, especially WBRT, often experience radiation-related side effects. Eric L Chang reported that patients treated with SRS combined with WBRT were at a greater risk of a significant decline in cognitive function compared with patients that received SRS alone [6]. The Radiation Therapy Oncology Group (RTOG) 0212 [7] and 0214 [8] trials have demonstrated that WBRT without avoidance of the hippocampus increased cognitive impairment by 3 times at 6 and 12 months after WBRT, which is significantly higher than the placebo group.

The neural pluripotent stem cells are mainly distributed in the hippocampus, which has been increasingly recognized as a common casualty of radiation-related damage in recent years [9]. Multiple studies have documented that WBRT could cause damage to the hippocampus and affect the formation of learning and memory associated with cognitive disorders [10]. The RTOG 0933 study revealed that avoidance of the hippocampus during WBRT resulted in preserving memory and quality of life as compared with historical series [11].

Accurate delineation of hippocampus is very important for the success of radiation treatment planning and hold the promise of reducing the radiation-related side effects [12, 13]. However, the delineation of the hippocampus is often a time-consuming and difficult task in clinical practice, due to its small volume and diffuse boundary. Furthermore, the individual experience of the radiation oncologists and the varied quality of the images complicates the situation even further. Recently, deep learning approaches based on convolutional neural networks (CNNs) have been widely investigated in the procedures of target delineation and has showed promising results for gross tumour volume, organs at risk, etc.[14, 15]. Therefore, auto-delineation of the hippocampus could be advantageous with the development of an artificial intelligence (AI) tool based on deep learning. It could not only meet the clinical needs of precise delineation, but also greatly reduce the time required for delineation.

The purpose of our study is to construct an AI tool to auto-delineate the hippocampus and validate the delineating accuracy and clinical feasibility.

## Materials and methods

**Fig. 1:**
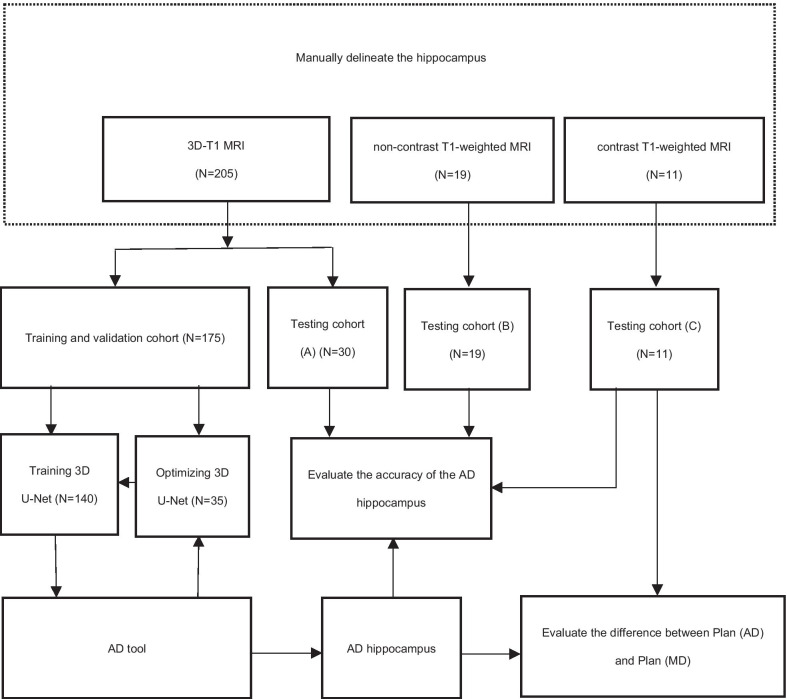
Outline of the study methodology. Here, *MD* represents manual delineation, *3D-T1* represents three-dimensional gadolinium contrast-enhanced T1-weighted, *MRI* represents magnetic resonance images, *AD* represents automatic delineation, *Plan (AD)* represents the generated radiotherapy plans using the AD hippocampus, and *Plan (MD)* represents the generated radiotherapy plans using the MD hippocampus

The entire study includes the selection of MR images, construction and optimization of the auto-delineate tool, and verification of the AD results (Fig. 1).

### Magnetic resonance images

We retrospectively collected 205 three-dimensional T1-weighted (3D-T1) sequence non-gadolinium contrast-enhanced MR images with an axial slice thickness of 1 mm from the First People’s Hospital of Hangzhou between January 2019 and June 2019. The data set was then randomly assigned to two cohorts: 140 cases were used to construct the AD tool and 35 cases were used to optimize its hyperparameters, and the remaining 30 cases were assigned to a testing cohort a. Considering that 3D-T1 MRI is not routinely used in clinical practice, we also collected external cases with non-3D-T1 MRI to test the performance of this AD tool. This includes 19 non-3D T1-weighted non-gadolinium contrast-enhanced MRIs with an axial slice thickness of 3 mm from Shanghai Chest Hospital between July 2019 and September 2019 (testing cohort b) and 11 non-3D T1-weighted gadolinium contrast-enhanced MRIs with an axial slice thickness of 1 mm from Hangzhou Cancer Hospital between July 2019 and September 2019 (testing cohort c). All the cases were > 18 years old, and the MRIs confirmed with normal hippocampus has not been violated by any disease. The MRI Machine Vendor in train cohort and testing cohort a is GE 3.0 T Signa. In testing cohort b and c is Siemens 1.5T Aera.

### Manual delineate (MD)

A total of 235 cases were manually delineated on the axial MRI slice by slice. The process of delineation referred to the hippocampus atlas contouring proposed by RTOG [16]. The delineation of the hippocampus was performed by one radiation oncologist (K.C.P), who was well-trained, especially in hippocampal delineation. Then, the results of delineation were reviewed and modified by an expert radiation oncologist (B.X) and a radiologist specializing in MRI imaging (Z.X.D).

### Network architecture

**Fig. 2:**
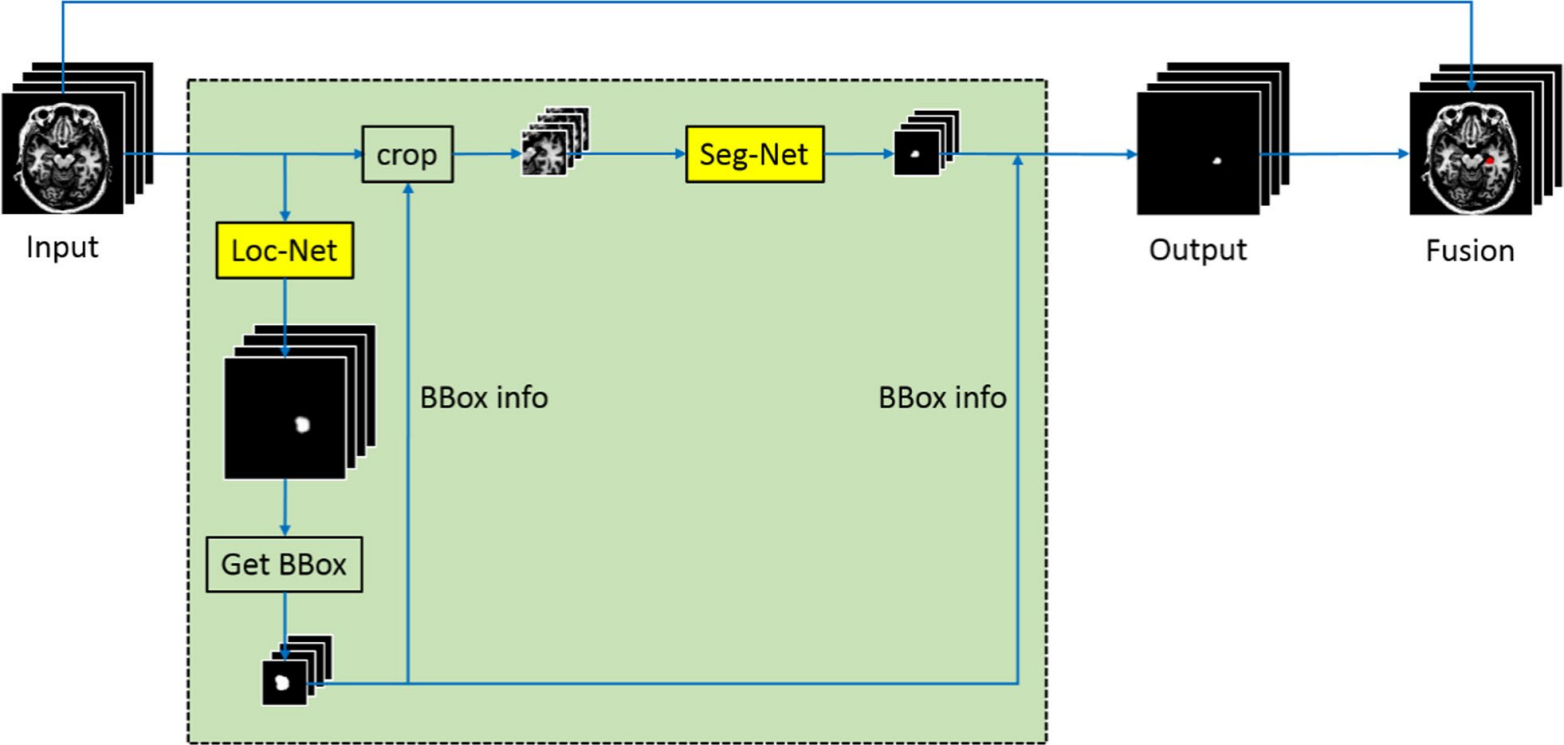
Network architecture in our work

We used the 175 3D-T1 MRI data sets to construct a three-dimensional (3D) CNN architecture to AD the hippocampus. The 3D CNN architecture we used is illustrated in Fig. 2. Before that, we will pre-process the images. The original axial pixel is 256 × 192, using those for algorithm training will occupy a large amount of graphics card memory, which is not conducive to algorithm training so we resized the whole slice pixel to 96 × 96, then subtracted the average value from the image and divided by the variance, the corresponding labeled image is divided by 255 and transformed to the 0–1 interval.

This 3D CNN contains 2 networks; a Loc-Net was used to locate the position of the hippocampus and a Seg-Net was used to delineate the hippocampus accurately. Both Loc-Net and Seg-Net were based on the 3D variants of U-Net [17], combined with Residual [18] and Attention gate blocks [19] without modification. The training and optimizing procedure of Loc-Net and Seg-Net is shown in Additional file 1: Fig. S1. After the training is completed, the image was also post-processed. We multiply the obtained result by 255 and binarize threshold processing, then we scale to the size of the original image, finally the contour line was extracted.

### Analysis of variation in contouring

We used the Dice similarity coefficient (DSC) and Average Hausdorff Distance (AVD) to evaluate the performance of our constructed AD tool in the three testing cohorts.

The DSC is the most used metric in measuring the overlap between two contours. The larger the DSC value is, the higher the similarity is between two contours. The DSC is defined as

$$DSC = \frac{{2\left| {S_{g} \cap S_{t} } \right|}}{{\left| {S_{g} } \right| + \left| {S_{t} } \right|}}$$

where $S_{g}$ is the ground truth delineation and $S_{t}$ is the delineation being evaluated [20].

Because the volume of the hippocampus is small, even small differences in delineation will have a significant impact on the DSC values [21]; we used AVD to compensate for this deficiency. AVD is the average distance between contours. The smaller the AVD value is, the higher the similarity is between two contours. AVD is defined as

$$AVD\left( {A,B} \right) = \max \left( {d\left( {A,B} \right),d\left( {B,A} \right)} \right)$$

where $A$ and $B$ are two finite point set and $d\left( {A,B} \right)$ is the directed AVD that is given by $d\left( {A,B} \right) = \frac{1}{N}\sum\limits_{a \in A} {\mathop {\min }\limits_{b \in B} \left\| {a - b} \right\|}$ [20].

### Generating radiotherapy plans

To evaluate whether the results of the AD hippocampus could be applied to clinical practice directly, we used 11 cases in testing cohort c to generate radiotherapy plans. All of the 11 cases had CT images scanned in the same position as MR images with a 1 mm slice thickness. The MR images were fused with the CT images and the hippocampus and HC-PRV on the MRI were copied to the CT images to generate radiotherapy plans. We defined the target volumes (TVs) as follows: the hippocampus was expanded by 5 mm in all three dimensions to form the planning risk volume (HC-PRV), the clinical target volume (CTV) was defined as the whole-brain, and the planning target volume (PTV) was defined as CTV expanded by 5 mm in three dimensions excluding the HC-PRV.

**Fig. 3:**
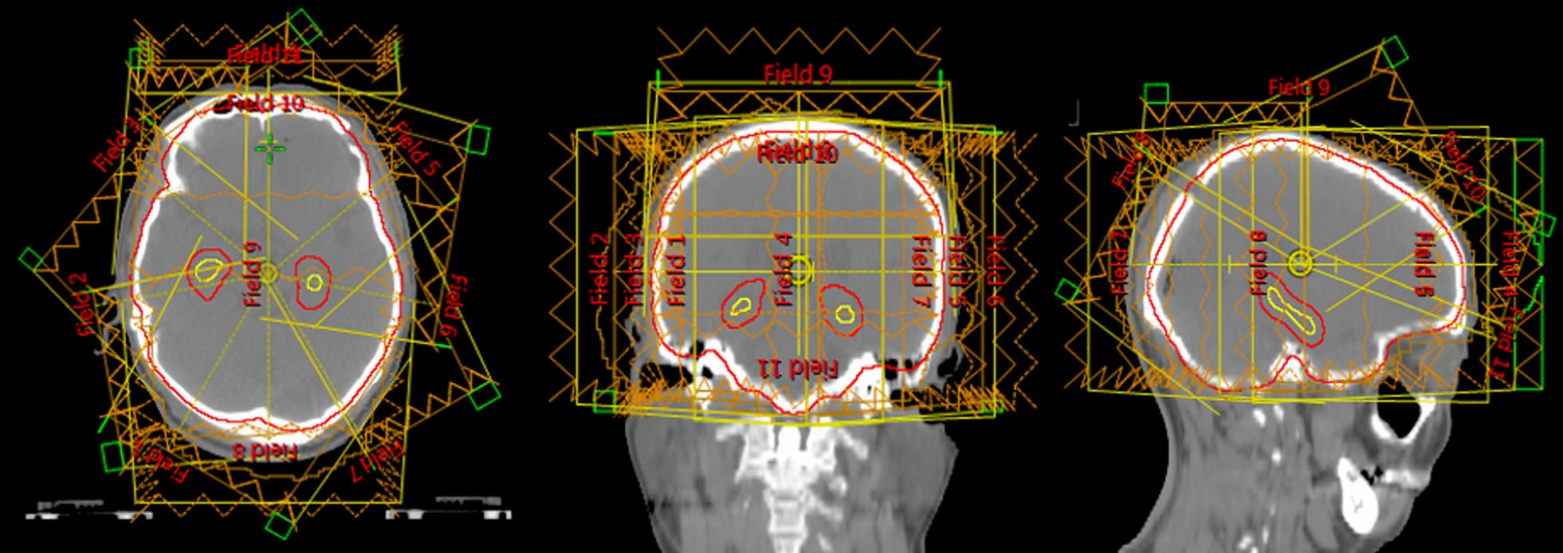
Distribution of the radiation field. Hippocampus (yellow line); Hippocampus planning risk volume (red line). 7-coplanar field (beam 1–7) and 4-non-coplanar field (beam 8–11) arrangement was used

Radiotherapy plans were made on Varian Eclipse Treatment Planning (Varian Medical System, USA) using the Intensity Modulated Radiation Therapy (IMRT) technique. The radiation field distribution is listed in detail in Additional file 1: Table S1, and a 7-coplanar field and 4-non-coplanar field arrangement was used for the IMRT plan (Fig. 3). The radiation dose of 10 × 3 Gy was prescribed to 95% of the PTV (V30Gy ≥ 95%) and the optimization parameters are listed in Additional file 1: Table S2. Dose constraints of normal tissue were referred to the RTOG 0933 protocol. Briefly, the mean dose of the hippocampus could not exceed 9 Gy, and the maximal hippocampal dose could not exceed 16 Gy. Both the AD and MD hippocampus were used to generate radiotherapy plans recorded as Plan (AD) and Plan (MD), respectively.

### Evaluation of radiotherapy plan

We used the MD hippocampus as the reference standard to evaluate the accuracy of the AD hippocampus, so we compared the differences of dose distribution between Plan (AD) and Plan (MD) based on the MD hippocampus. For PTV, we evaluated the dose received at 2% (D2%), D98%, V30Gy, and maximum dose (D_max_). For hippocampus, we evaluated the D_max_, mean dose (D_mean_), and V16Gy. For the lens we evaluated Dmax.

### Time consuming analysis

The time spend in the AD and MD delineation of the hippocampus was recorded and compared in testing cohort a.

### Statistical analysis

Paired Student’s *t* tests were used to compare the volume of the hippocampus, DSC and AVD of the hippocampus and HC-PRV between AD and MD and the dose-volume parameter variation between Plan (AD) and Plan (MD). Independent sample *t* tests were used to compare DSC and AVD between the three testing cohorts. All analyses were performed using SPSS version 23 (SPSS, New York 10504-1722 United States). *P* < 0.05 was considered significant.

## Results

### Patient characteristics

The characteristics of the training cohort and testing cohort are shown in Table 1.

**Table 1:** Basic characteristics of the 235 patients

	Training cohort (n = 175)	Testing cohort a (n = 30)		Testing cohort b (n = 19)		Testing cohort c (n = 11)	
		AD	MD	AD	MD	AD	MD
Number of male patients (percentage)	79 (45.1)	14 (46.7)		17 (89.5)		7 (63.6)	
Median age(range)	68 (36–98)	69.5 (35–88)		63 (46–77)		64 (55–82)	
Volume of hippocampus (mean ± SD cm^3^)	4.71 ± 1.03	4.82 ± 0.91	4.85 ± 0.97	6.87 ± 0.81	4.95 ± 0.55	4.85 ± 0.80	4.64 ± 0.76
*P* value		> 0.05		< 0.05		> 0.05	

Testing cohort a: 3D T1 MRI with 1-mm slice thickness; testing cohort b: T1-weighted MRI with 3-mm slice thickness; testing cohort c: T1-weighted MRI with 1-mm slice thickness

There was no statistical difference between the MD and AD hippocampal volume in testing cohort a (*P* = 0.791) and cohort c (*P* = 0.430). However, the AD hippocampus is larger compared with that of MD in the testing cohort b (*P* < 0.01). The age and sex were well balanced in these cohorts.

### Delineation consistency

**Fig. 4:**
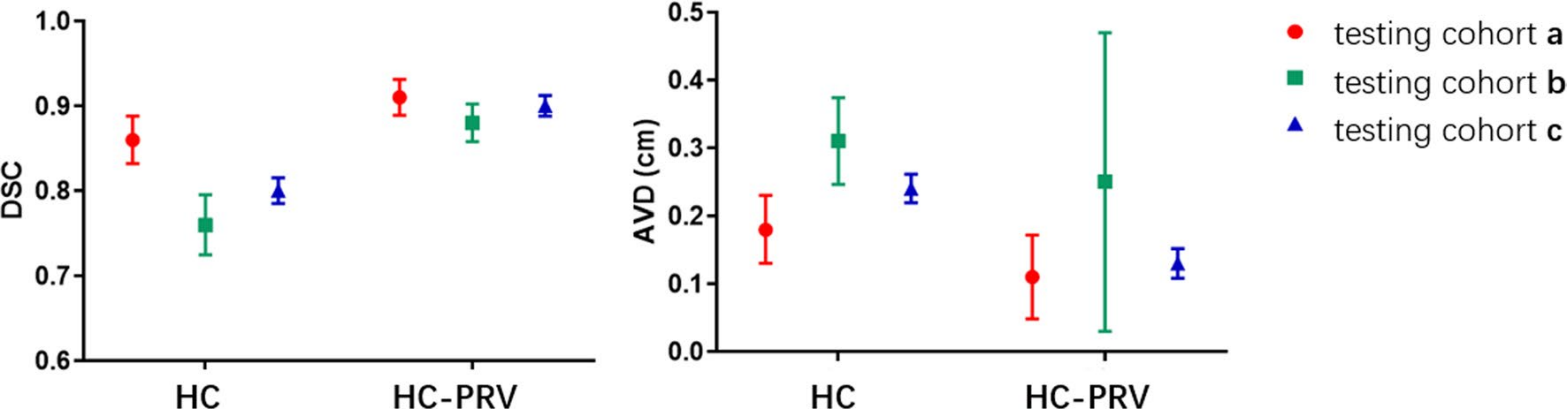
Mean AVD and DSC values with error bars for hippocampus and HC-PRV in the three testing cohorts. Note: testing cohort a: 3D T1 MRI with 1-mm slice thickness; testing cohort b: T1-weighted MRI with 3-mm slice thickness; testing cohort c: T1-weighted MRI with 1-mm slice thickness; HC: hippocampus; HC-PRV: planning risk volume of the hippocampus

The values of DSC and AVD of the three cohorts are shown in Fig. 4.

The mean DSC of the hippocampus and HC-PRV were 0.86 ± 0.028 and 0.91 ± 0.021 in testing cohort a; 0.76 ± 0.035 and 0.88 ± 0.022 in cohort b; 0.80 ± 0.015 and 0.90 ± 0.012 in cohort c, respectively. The DSC of the hippocampus in cohort a was higher than those in cohort b and cohort c (*P *< 0.01). The DSC of the hippocampus in cohort c is higher than that in cohort b (*P* = 0.005). However, there was no statistical difference in DSC of the HC-PRV (*P* = 0.11) in these cohorts. In addition, DSC of HC-PRV was higher than that of the hippocampus in all three cohorts (*P* < 0.01).

The mean AVD of the hippocampus and HC-PRV were 1.8 ± 0.50 mm and 1.1 ± 0.62 mm in testing cohort a; 3.1 ± 0.64 mm and 2.5 ± 2.2 mm in cohort b; 2.4 ± 0.21 mm and 1.3 ± 0.22 mm in testing cohorts c, respectively. The AVD of the hippocampus and HC-PRV in testing cohort a were smaller than those in cohort c (*P *< 0.01). Both are smaller than in cohort b (*P* < 0.05). The AVD of HC-PRV is smaller than the AVD of hippocampus (*P* < 0.01) except in testing cohort b (*P* = 0.15).

### Dosimetric results

**Fig. 5:**
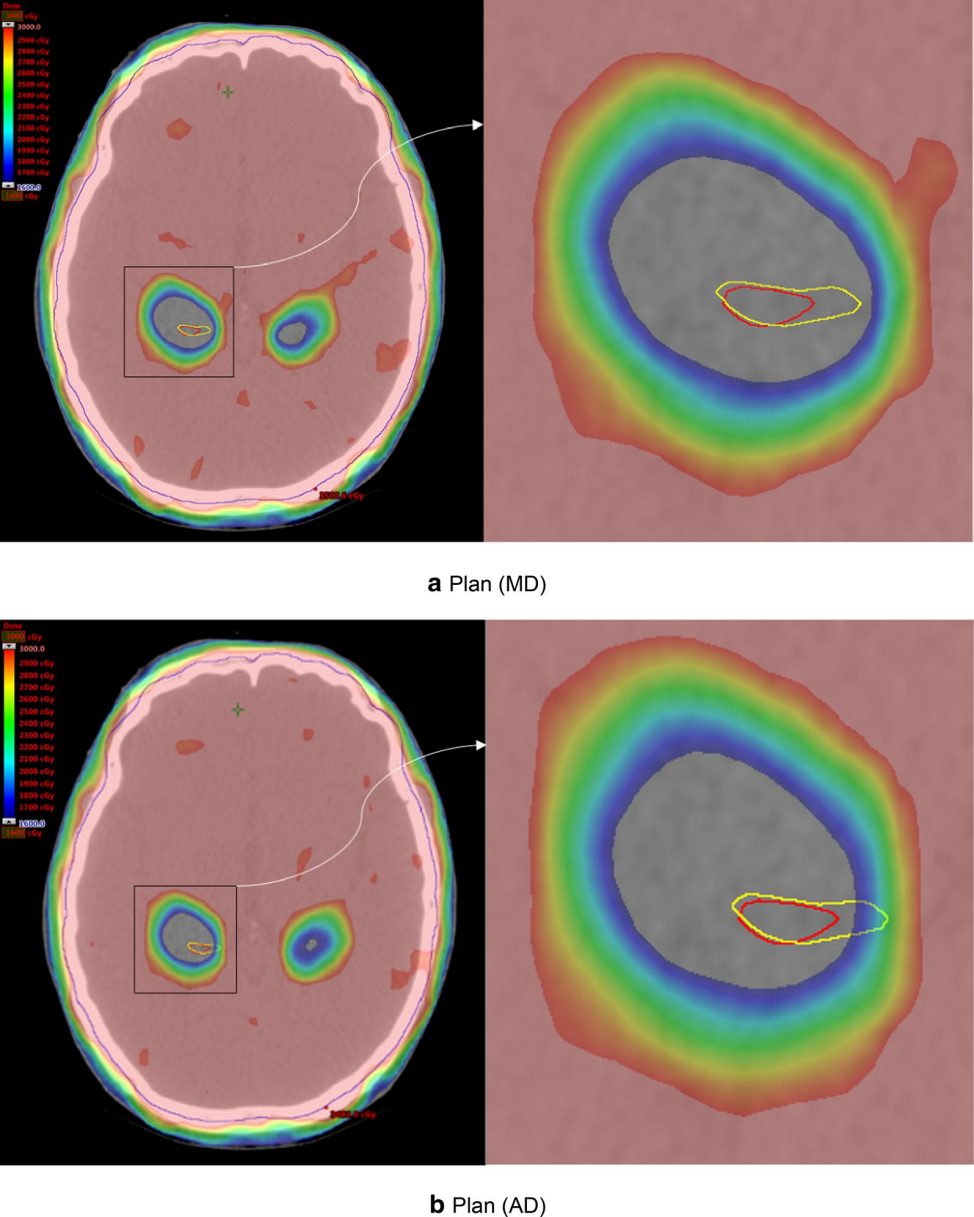
Variations of dose distributions between plan (MD) and Plan (AD). **a** Plan (MD), **b** Plan (AD). Manually delineated hippocampus (yellow line), automatically delineated hippocampus (red line), and PTV (blue line). Radiotherapy plan generated by **a** manually and **b** automatically delineated hippocampus. Both plans were evaluated using the AD hippocampus. In Plan (AD), a small area of the manually delineated hippocampus received a dose of more than 1600 cGy

The dose parameters of Plan (AD) and Plan (MD) are listed in Table 2. Figure 5 presents the dose distribution of Plan (AD) and Plan (MD) for a case. For the hippocampus, the average D_mean_ was 906.9 ± 17.2 cGy in Plan (AD) and 898.8 ± 14.1 cGy in Plan (MD). Although the difference was statistically significant, it was not considered clinically significant. There are 6 cases in Plan (AD) which had a D_max_ of the hippocampal exceeding 1600 cGy (range 1624.2–2196.2 cGy), the V16Gy of the hippocampus in these 6 cases ranged from 0.002 to 0.187%. For all the cases, D_max_ of the hippocampal in Plan (MD) did not exceed 1600 cGy. For PTV and lens in these 11 cases, the differences were not statistically significant and all RT plans met the dose constraints.

**Table 2:** Dosimetric comparison between Plan (AD) and Plan (MD)

		Plan (AD) (mean ± SD)	Plan (MD) (mean ± SD)	Plan deviation (mean ± SD)	*P* value
PTV	D_max_ (cGy)	3528.3 ± 24.4	3531.8 ± 21.9	− 3.55 ± 16.6	0.513
	V30 (%)	94.96 ± 0.14	95 ± 0	− 0.04 ± 0.14	0.423
	D98 (cGy)	2519.1 ± 61.2	2540.9 ± 24.2	− 21.9 ± 53.2	0.223
	D2 (cGy)	3386.2 ± 11.4	3390.7 ± 9.7	− 4.4 ± 7.2	0.079
Hippocampus	D_mean_ (cGy)	906.9 ± 17.2	898.8 ± 14.1	8.1 ± 11.2	0.046
	D_max_ (cGy)	1671.5 ± 264.7	1450.3 ± 62.1	221.2 ± 257.9	0.022
	V16(%)	0.046 ± 0.070	0 ± 0	0.046 ± 0.070	0.064
Lens	D_max_ (cGy)	567.5 ± 42.0	568.5 ± 47.0	− 1.03 ± 8.56	0.712

Plan deviation = Dose in Plan (AD)-Dose in Plan (MD); V30: the volume of PTV getting 30 Gy; D98: the dose received at 98% of PTV; D2: the dose received at 2% of PTV; V16: the volume of hippocampus getting 16 Gy

### Analysis of delineation time

The median time required to AD the hippocampus on both sides of these 30 cases is 9.35 s (range 8.49–10.0 s). This result is significantly less than the time required for MD (*P *< 0.001), which is 480 s (range: 436–542 s).

## Discussion

Automatic delineation of TVs based on AI has become a hot research area in recent years, including semiautomatic tool like atlas-based auto-segmentation (ABAS) and fully automated based contouring. ABAS refers to the propagation of segmented structures from atlas images onto a patient image data set using deformable image registration (DIR). It is susceptible to topological errors which result in lower accuracy than MD for small TVs. Considering that the volume of the hippocampus is very small, large errors may occur when using ABAS. However, the AI-based AD technology uses deep neural network architectures with multiple (2 or more) hidden layers (those between input and output layers) to learn features from a dataset by modeling complex nonlinear relationships. When delineating hippocampus, AI-based AD technology has advantages over ABAS.

However, few studies have investigated the ability of AI-based AD hippocampus based on MRI. In this study, we constructed a hippocampal AD tool based on 3D T1 MRI images using 3D CNN, the performance of which was tested in three independent cohorts. The final results showed that the consistency between AD and MD hippocampus was acceptable, and that the difference in the treatment planning based on HC-PRV could be negligible, which indicates that the AD approach has the potential of clinical application for hippocampus segmentation. In addition, unlike ABAS, which mostly needs to be manually modified after the AD is completed, the hippocampus AD by this tool can be applied without manual modification, which greatly reduces the time spend for delineation. The delineating time has been reduced from nearly 4 min for MD to less than 10 s for AD.

3D-T1 MRI has been recommended as the standard modality for use in hippocampal delineation according to the RTOG 0933 protocol. Considering that 3D-T1 MRI is not regularly used in clinical practice, we also included non-3D T1-weighted MRI images when testing the cohorts to evaluate the expansibility of the tool. As expected, cohort a showed the best consistency between the AD and MD hippocampi, and the results of cohort b were the worst. Additionally, the volume of the AD hippocampus in cohort b was the largest in all of the delineated hippocampi. Since only the 3D-T1 cases were selected as the training cohort, this may be one of the reasons. If we trained our model with images acquired as in cohort b and c, the results of cohorts b and c may improve. But the more important reason for such a disparity might be related to the axial slice thickness of MRI; improving the slice thickness of 3 mm in cohort b will cause blurring in the 2D slice because of the partial volume effect and the decreased spatial resolution, which leads to inferior consistency between the AD and MD hippocampi and the large error bar in cohort b. In terms of the gadolinium enhancement, since it will not have any impact on the hippocampus and its surrounding tissues [22], it will not affect the results of the delineation. In addition, although the same slice thickness of 1 mm was used in cohorts a and c, the DSC and AVD in cohort a were improved compared to those of cohort **c**, which might be explained by the different image quality due to the scan sequence. With 3D acquisition, due to their higher signal to noise ratio and isotropic voxel size, maximal intensity projection (MIP) reformation is allowed in arbitrary planes, which contributes to good discrimination between grey and white matter [23]. Actually, the hippocampus was most easily identified in the 3D T1 image. According to this study, our AI tool performed better in 3D-T1 MRI, and we are enthusiastic to maximize its utility.

Although there were geometric differences between the AD and MD hippocampi, those differences may not reflect on the following formation of the HC-PRV and radiation treatment plans. The mean DSC and AVD of hippocampus in those 60 MRI data sets are 0.82 and 2.3 mm (in 3D-T1 MRI they are 0.86 and 1.8 mm), respectively. For HC-PRV, the mean DSC and AVD are 0.90 and 1.6 mm (in 3D-T1 MRI they are 0.91 and 1.1 mm), respectively. It can be seen that the volume of the hippocampus is small and therefore more sensitive to the overlap of location. Since the HC-PRV was formed by expanding the hippocampus by 5 mm in all three dimensions, the performance of the HC-PRV was better than that of the hippocampus because of the relatively large volume. Furthermore, we compared the dosimetric differences between Plan (AD) and Plan (MD) to evaluate the clinical feasibility. Since the dose received by the MD hippocampus represents the dose that the patient’s hippocampus may receive during the actual application, we compared the differences of dose distribution between Plan (AD) and Plan (MD) based on the MD hippocampus. As shown in Table 2, the dose volume constraints of hippocampus in Plan (AD) did not agree well with those in in Plan (MD), mainly because the evaluation of plan (AD) was based on the manually delineated hippocampus. However, as is shown in Fig. 5, all the V16Gy of the hippocampus in Plan (AD) were very small, so that only 0.0082 cm^3^ were at maximum. According to the study of Paul D. Brown, it is considered clinically acceptable when the V16Gy of the hippocampus is less than 0.03 cc [24]. In terms of D_mean_, according to the RTOG 0933 protocol, it is still within the acceptable range when the D_mean_ of the hippocampi does not exceed 10 Gy. And all RT plans were within this acceptable range, although there was some difference in Plan (AD) and Plan (MD). For PTV, since the volume was much larger than that of the hippocampus, the geometric differences of the hippocampal volume have little effect on the dose coverage of PTV, and there was no significant difference in the dosimetric parameters of PTV between Plan (AD) and Plan (MD).

There are limitations to this study, including those that are inherent to retrospective studies, such as unknown selection bias. The images used to construct the hippocampal AD tool were from a uniform dataset with high quality and good contrast which, to some extent, limited the tool's extensibility. Although there was a geographical loss in inferior images in the validation process, dosimetric analysis did not differ significantly in terms of treatment planning, indicating that the AD tool has potential for clinical application. Another issue that needs to be mention is the question of the MD hippocampus set as the golden standard. It has been reported that even for the same target delineated by different radiotherapists, DSC may reach 0.6–0.8 [25]. In this study, the delineation of the hippocampus was performed by only one radiation oncologist to minimize differences between different radiotherapists. In light of this, for the delineation of hippocampus that could not be easily distinguished on images, the AD tool based on deep learning has natural advantages over manual delineation, which is more conducive to maintaining consistency in clinical practice or prospective trials.

## Conclusions

The automatic delineation of the hippocampus based on a deep learning algorithm achieved satisfactory results, which could have a positive impact on improving delineation consistency without significantly impacting dose parameter and reducing work load in clinical practice.

## Supplementary Information

**Additional file 1**. Supplementary figures and tables.

## Data Availability

The datasets used and analyzed during the current study are available from the corresponding author on reasonable request.

## Abbreviations

WBRT: Whole brain radiotherapy
CNNs: Convolutional neural networks
AD: Automatic delineation
MD: Manually delineated
AI: Artificial intelligence
RT: Radiotherapy
DSC: Dice similarity coefficient
AVD: Average Hausdorff Distance
SRS: Stereotactic radiosurgery
RTOG: Radiation Therapy Oncology Group
3D-T1: Three-dimensional T1-weighted
TVs: Target volumes
HC: Hippocampus;
PRV: Planning risk volume
CTV: Clinical target volume
PTV: Planning target volume
IMRT: Intensity Modulated Radiation Therapy
MIP: Maximal intensity projection
