# Supplementary material for: Deep learning-based automatic delineation of the hippocampus by MRI: geometric and dosimetric evaluation
Source: Radiat Oncol. 2021 Jan 14;16:12. doi: 10.1186/s13014-020-01724-y (PMC7807715; doi:10.1186/s13014-020-01724-y)
Supplement: Supplementary file 1 — Additional file 1. Supplementary figures and tables. [file 13014_2020_1724_MOESM1_ESM.docx]

**Supplementary**

(a)Loc-Net training procedure (b) Seg-Net training procedure

**Fig. S1. Training and optimizing procedure of Loc-Net and Seg-Net.**

**Table S1. Beam arrangement**

| beam | Varian Scale Couch Angle (degrees) | Varian Scale Gantry Angle (degrees) |
| --- | --- | --- |
| 1 | 0 | 210 |
| 2 | 0 | 260 |
| 3 | 0 | 310 |
| 4 | 0 | 0 |
| 5 | 0 | 50 |
| 6 | 0 | 100 |
| 7 | 0 | 150 |
| 8 | 90 | 210 |
| 9 | 90 | 270 |
| 10 | 90 | 330 |
| 11 | 90 | 20 |

**Table S2. Planning algorithm constraints**

| Structure | LINAC-Based IMRT Plan Criteria | weight |
| --- | --- | --- |
| PTV | Max dose:3250 cGy | 100 |
|  | Lower dose:3050 cGy | 150 |
| hippocampus | Max dose:1000 cGy | 200 |
|  | Mean dose:550 cGy | 115 |
| lens | Max dose:300 cGy | 100 |
